# Mapping Histological Slice Sequences to the Allen Mouse Brain Atlas Without 3D Reconstruction

**DOI:** 10.3389/fninf.2018.00093

**Published:** 2018-12-11

**Authors:** Jing Xiong, Jing Ren, Liqun Luo, Mark Horowitz

**Affiliations:** ^1^Department of Electrical Engineering, Stanford University, Stanford, CA, United States; ^2^Department of Biology, Howard Hughes Medical Institute, Stanford University, Stanford, CA, United States

**Keywords:** nonrigid, image registration, Markov random field, histological images, 2D to 3D, Allen Mouse Brain Atlas, histogram of oriented gradients

## Abstract

Histological brain slices are widely used in neuroscience to study the anatomical organization of neural circuits. Systematic and accurate comparisons of anatomical data from multiple brains, especially from different studies, can benefit tremendously from registering histological slices onto a common reference atlas. Most existing methods rely on an initial reconstruction of the volume before registering it to a reference atlas. Because these slices are prone to distortions during the sectioning process and often sectioned with non-standard angles, reconstruction is challenging and often inaccurate. Here we describe a framework that maps each slice to its corresponding plane in the Allen Mouse Brain Atlas (2015) to build a plane-wise mapping and then perform 2D nonrigid registration to build a pixel-wise mapping. We use the L2 norm of the histogram of oriented gradients difference of two patches as the similarity metric for both steps and a Markov random field formulation that incorporates tissue coherency to compute the nonrigid registration. To fix significantly distorted regions that are misshaped or much smaller than the control grids, we train a context-aggregation network to segment and warp them to their corresponding regions with thin plate spline. We have shown that our method generates results comparable to an expert neuroscientist and is significantly better than reconstruction-first approaches. Code and sample dataset are available at sites.google.com/view/brain-mapping.

## 1. Introduction

**Figure 1:**
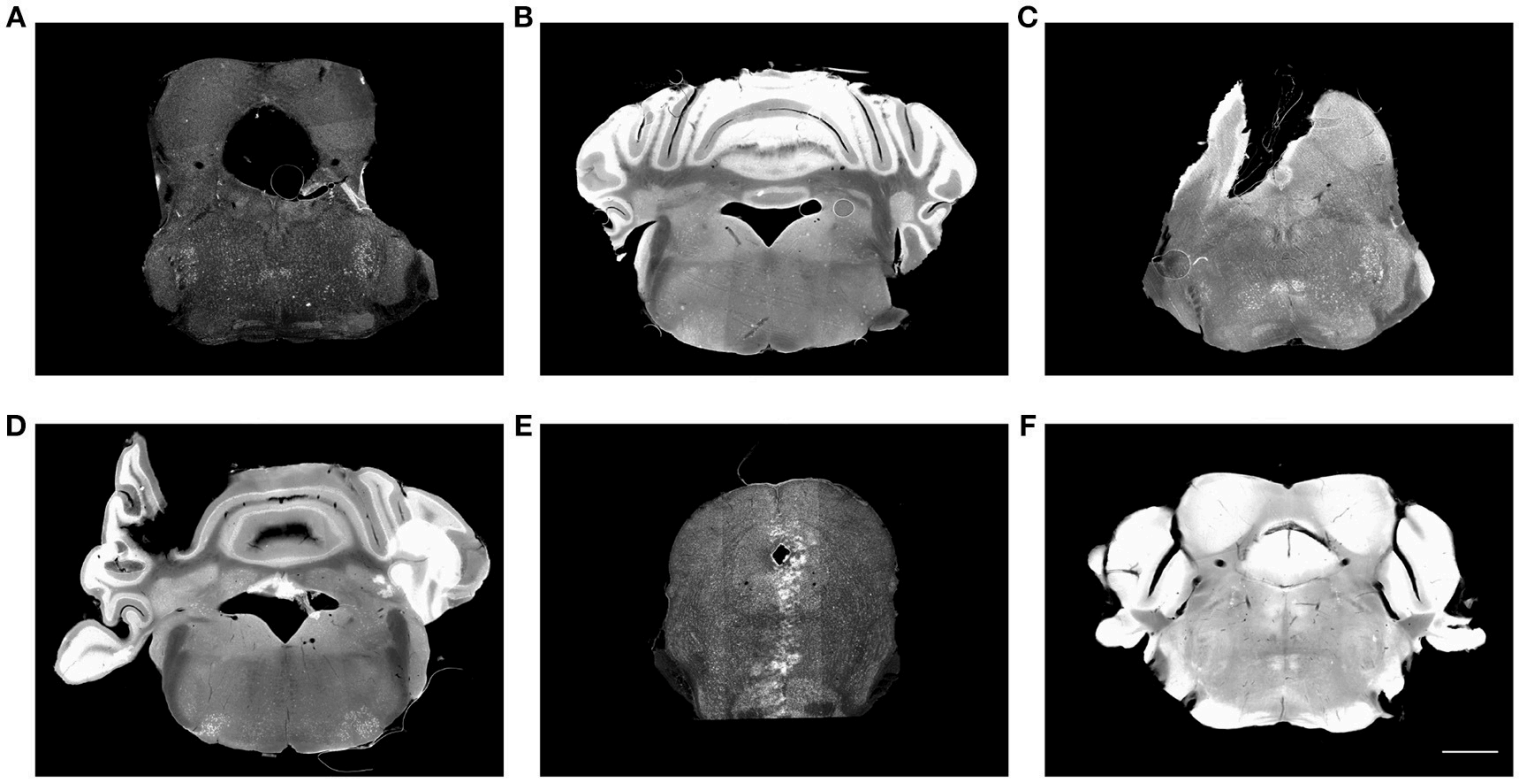
Histological images suffer from multiple artifacts. **(A)** Enlarged aqueduct, tear, stitching artifact. **(B)** Uneven staining, air bubble. **(C)** Missing tissue, air bubble, uneven staining. **(D)** Misplaced tissue, fold, uneven staining. **(E)** Missing tissue, stitching artifact, imaging artifact. **(F)** Over staining. Scale, 1 mm.

Neuroanatomical studies have traditionally been performed in histological sections, followed by manually annotating data based on histological stains in comparison with a brain atlas. For large-scale analyses, this procedure is labor-intensive, time-consuming, variable and sometimes subjective. It is crucial to standardize and digitalize anatomical information to allow data from multiple brains to be compared in the same reference brain. To this end, detailed anatomical brain reference atlases have been established for both human and animal model studies (Lein et al., 2007; Hawrylycz et al., 2012; Bakker et al., 2015; Tiesinga et al., 2015). Ideally, all experimental brain images would be automatically registered to an anatomical reference volume, creating a platform for the comparison and integration of results from different experiments. However, registering laboratory histological images to an atlas is still challenging in terms of accuracy, universality, and time-efficiency. One of the major issues is that brain histological data sets often suffer from artifacts, such as enlarged ventricles (holes), missing tissue, folding, air bubbles, uneven staining, tears, and slice-independent distortions, shown in Figure 1. Existing programs mapping a 2D histological sequence to a reference volume often require an initial reconstruction from these partially corrupted slices and therefore only work well with datasets of very good quality. However, histological datasets often require months of experiments to generate results. Most labs still rely on manual brain region identification to fully utilize all of the experimental slices even if they are partially corrupted. This labor-intensive and time-consuming approach is highly variable and subjective among researchers.

**Figure 2:**
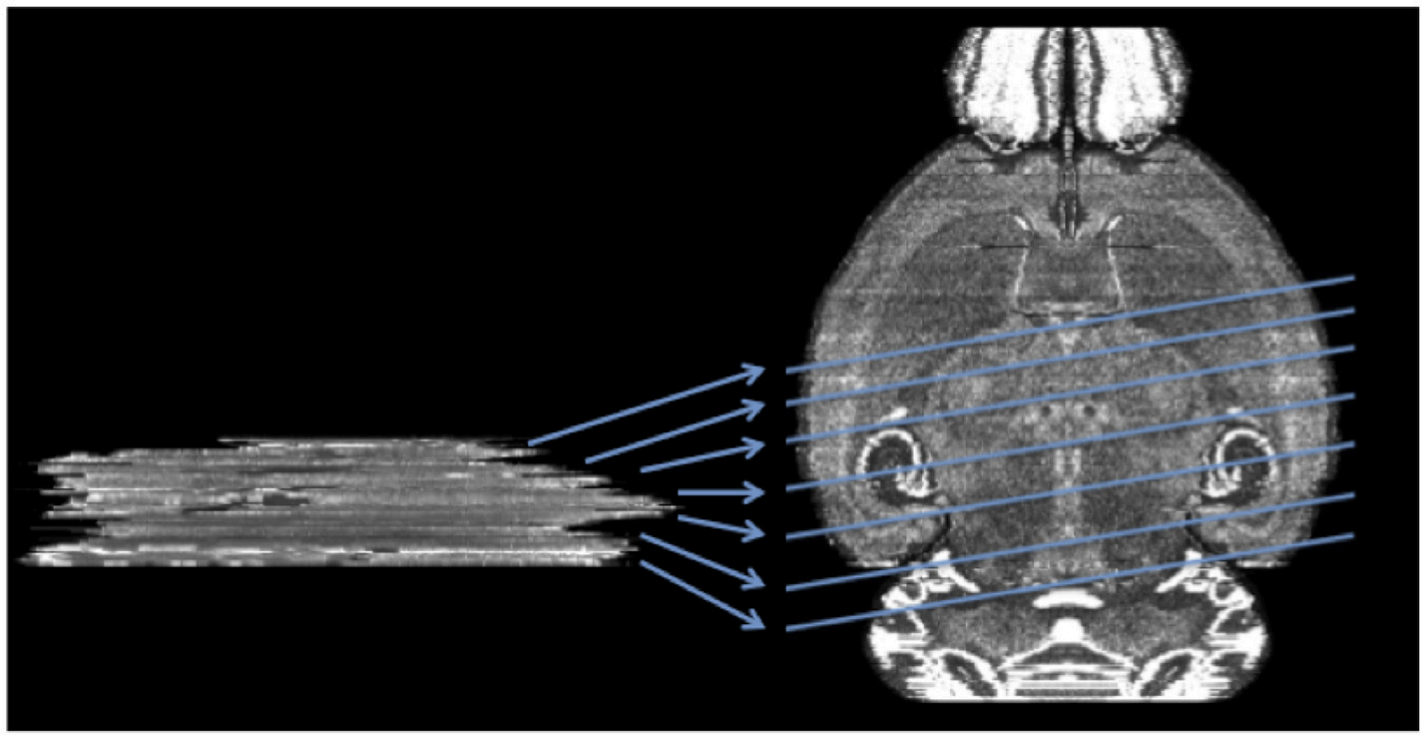
Mapping a sequence of histological mouse brain slices to the atlas volume of ABA (horizontal view of coronal sections). Left side shows a real histological stack. Right hand side is the ABA.

In this work, we introduce a method to register a sequence of coronal histological sections of mouse brain to the grayscale Nissl volume of the Allen Mouse Brain Atlas (2015) (ABA) (Lein et al., 2007; Allen, 2015) by first identifying the matching sectioning plane in the atlas volume for each slice and then performing 2D nonrigid registration. The general idea and an example dataset are shown in Figure 2.

The problem of mapping a sequence of histological slices to a reference brain has been well studied (Pichat et al., 2018). This prior work first reconstructs an initial volume estimate from the slices and then registers this reconstructed volume to the reference. Some work focuses on the reconstruction problem because registration between a reconstructed volume and a reference is relatively standard (Dauguet et al., 2007; Stille et al., 2013; Mertzanidou et al., 2017); other work discusses the reconstruction problem in the absence of reference volumes (Ourselin et al., 2001; Ju et al., 2006; Bagci and Bai, 2010; Cifor et al., 2011). Initial work using this approach reconstructed the experimental volume by pairwise registration of adjacent slices (Ourselin et al., 2001; Cifor et al., 2011; Stille et al., 2013). Due to tissue distortions, rigid registration is not sufficient. But pairwise nonrigid registration propagates any registration errors throughout the whole brain. This is especially problematic if any slice has a large deformation such as missing tissue.

To improve overall reconstruction results and reduce error propagation, some methods align each slice with multiple neighboring images. For example, Ju et al. (2006) reduced error propagation by warping each slice with a weighted linear and nonlinear combination of warp fields to multiple adjacent slices. Others used blockface images (Dauguet et al., 2007) or selected internal reference slices to reconstruct small chunks and then put together the entire volume (Bagci and Bai, 2010; Mertzanidou et al., 2017). However, with almost every slice at least slightly distorted, internal nonrigid registration will likely change the original shape of biological structures. Because this process tries to maximize the similarity between adjacent thin, e.g. 40 μm to 60 μm, histological slices, curved 3D structures along the sectioning direction may end up straightened. This 3D structure-straightening problem is known as the banana problem or the z-shift problem (Adler et al., 2014; Majka and Wójcik, 2016). Once this error is introduced, it is hard to reverse completely even when this volume is registered to the reference. To avoid these volume distortion errors, one needs to use the reference volume earlier in the process by registering each experimental slice to its corresponding sectioning plane in the reference volume.

Now the main challenge is finding the corresponding plane for each slice (Yang et al., 2012). This task is made more difficult because the experimental volumes have a non-standard sectioning angle, the brains are tilted in the sectioning machine and have an anisotropic resolution. The reconstructed volume has a very high resolution in the sectioning plane (determined by the resolution of the imaging system) and comparatively low resolution along the sectioning axis (limited by the minimum slice thickness). The slice-to-slice approaches usually assume that cutting planes are parallel to the acquisition planes of the 3D medical image (Pichat et al., 2018) or at least the cutting angle of the microtome are constant through the cutting process (Gibson et al., 2012; Yang et al., 2012; Goubran et al., 2013). Abdelmoula et al. (2014a) used one of the most prominent features—the hippocampus—to determine the best match plane for each experimental tissue section, however, the searching space is limited, and the cutting angle difference is not considered. Papp et al. (2016) developed an interactive tool with which neuroscientist can reslice a reference volume with adjustable angle and position. Five to ten slices are mapped, then the remaining slices are interpolated. Other recent work used an iterative approach by first reconstructing a small volume and registering these slices to their corresponding planes in the reference brain (Yang et al., 2012; Goubran et al., 2013). Yang et al. (2012) selected a reference slice that maximizes the normalized mutual information after a 2D rigid registration is performed between a histological slice and each MRI slice. Goubran et al. (2013) registered each histological slice of a human brain to its corresponding MRI slice after blocks of the human brain were registered. Possum (Majka and Wójcik, 2016) developed an open source software framework that reconstructs a volume with or without using an external reference. While these methods work well when the sectioning angle difference is small, they introduce errors at larger sectioning angles.

To avoid these issues, we concurrently estimate the sectioning angle difference and the best matching planes in the atlas volume for each slice. This approach requires us to find the best matching slice in the reference before applying nonrigid deformations. Since the resulting slice comparisons are noisy, we aggregate information from all slices and use information about the brain's structure to find the best match. Our method does not have a reconstruction step, therefore completely eliminating the z-shift problem. The details of our method are given in the next section.

After each matching reference slice has been determined, we need to perform a 2D registration between it and its matching histological slice. Free Form Deformation (FFD) (Rueckert et al., 1999; Rohlfing and Maurer, 2003) has been the most common approach in neuroscience studies to map histological brain images (Jefferis et al., 2007; Geha et al., 2008; Abdelmoula et al., 2014a,b; Dorocic et al., 2014; Verbeeck et al., 2014; Costa et al., 2016). Mutual information is often used as the similarity metric to register histological slices because of image appearance difference caused by acquisition procedure variability. This method is highly dependent on the initial condition because the choice of using mutual information as a similarity metric often leads to a highly non-convex optimization problem with many local minima (Haber and Modersitzki, 2006). Because of staining variability within a slice, using mutual information does not always work.

Instead, we find that the L2 norm of histogram of oriented gradients (HOG) (Dalal and Triggs, 2005) difference suits histological slice properties better. Because HOG is non-differentiable, we base our work on the elegant discrete Markov random field (MRF) approach in Glocker et al. (2008). Based on the tissue labeling information of the annotation volume of ABA, we build a MRF model based on tissue coherency. To better deal with the data-specific properties of our experimental dataset—the heavily deformed ventricular systems—we make further improvement including segmenting a biological structure and warping them with thin plate spline (TPS) (Bookstein, 1989).

Our strategy makes the maximum use of the reference volume, successfully deals with the non-standard sectioning angle problem, preserves the curvature of the object—eliminating the z-shift problem (Adler et al., 2014), and is more tolerant to data corruption. This method takes into account some of the brain's structural properties to minimize error, including the compressibility of different brain regions. The algorithm is tested both on the full brain and sectional brain data, yielding faster and better correspondence than possible before.

## 2. Material and Methods

This section describes in more detail how we find the sectioning angle difference and the best matching plane in the reference volume for each histological slice (Section 2.1) and nonrigidly register each slice to the corresponding sectioning plane in the atlas (Section 2.2).

Both in the 2D to 3D localization and the 2D nonrigid registration steps, a relatively sensitive and quantitative similarity measure is needed. The state of art is to use normalized mutual information (Jefferis et al., 2007; Geha et al., 2008; Dorocic et al., 2014; Costa et al., 2016). Despite its wide use, it does not work well in our images since this metric fails when intra-slice uneven staining causes intensity variability within a structure, which breaks the statistical correlation between a slice and its target image. In addition, because each slice is at least slightly distorted, when measuring the similarity between a distorted experimental slice and an atlas slice, we need to either correct the distortions or find a metric that is friendly with distorted images. The former is hard because it is hard to constrain the deformation to only correct the distortions rather than making two images more similar than it should be. After being rotated, some curves and edges will be jagged. We therefore decided to find a metric that worked well even with the existence of distortions.

When searching for a better metric, we also wanted to find one that would work well for our images. Our image characteristics include:

1. Staining reagent and microscopic setting difference can cause direct comparison of intensities to be not useful. Even worse, due to the non-uniformly applied staining reagent, some slices are unevenly illuminated.
2. Nissl-stained (Glaser and Van der Loos, 1981) images only highlight the cell body of neurons. Two matching images will show corresponding anatomical structures but do not have pixel-wise cell body level correspondence.
3. Sparsely scattered or densely populated cell bodies make images low-contrast and noisy. Many descriptors that work with man-made scenes do not perform well.
4. Distortions caused by brain's elasticity require metrics that work even when the two images are slightly distorted from each other. This distortion tolerance also allows it to compare a distorted histological slice to a reference slice.
5. Even though the newest Nissl volume of the Allen Mouse Brain Atlas (2015) is smoother than the Allen Mouse Brain Atlas (2011), it is still Nissl-stained volume constructed from physically-sectioned mouse brain slices and is not perfectly aligned. So an ideal metric should be somewhat tolerant to this imperfect alignment.

As shown by Dalal and Triggs (2005), HOG has the capability to deal with pose, illumination, and background variations which mimic many of the issues in our images. This descriptor divides an image patch into small cells. In each cell it bins each pixel's gradient and forms a histogram. Each histogram is normalized based on the magnitude of the histograms of its neighboring cells in a local block. Each block is then described by the concatenation of these normalized histograms. HOG describes a small patch rather than individual pixels. Gradient binning gives some flexibility to distortions but still captures the overall direction of edges. It also well accommodates the unsmoothness nature of the atlas volume. Because even if an unsmooth volume is rotated, and edges are not perfectly aligned, true gradients are still kept. Normalizing a cell's magnitude by the magnitude of its neighboring cells reduces the negative effect from uneven staining. Therefore we use the L2 norm of HOG difference between two images as the similarity metric. To use this metric the two images first are brought to the same coordinates with a similarity transformation estimated with the Umeyama method (Umeyama, 1991) based on contour point correspondence generated by Shape Context (Belongie et al., 2000). Smooth tissue contours are extracted by applying the Fourier transform on the boundary curve and removing high-frequency components. To accommodate the global deformation caused by the force in the direction of sectioning, slices are further rescaled in horizontal and vertical directions. We then extract HOG features from both images and generate a scalar error by summing the squared difference between HOG feature vectors for each block at the same coordinate in the two images. For 2D to 3D localization, we use a large cell size so that the metric is less sensitive to local distortions. For 2D registration, we compute the nonrigid transformation that minimizes the HOG L2 difference with a small cell size.

### 2.1. 2D–3D Localization

Since histological slices are often cut with near constant angles with a microtome (Gibson et al., 2012; Yang et al., 2012; Goubran et al., 2013), it is fair to assume a constant cutting angle throughout the whole brain. Because the atlas is uniform in each dimension, to find the cutting angle difference, we rotate the atlas with different angles, resection it into coronal slices, reindex the slices in order, and compare the new resectioned atlas slices to the histological sequence.

The following sections give our dynamic programming formulation to solve the alignment problem to determine the slicing angle and a simple method to increase sensitivity to angular shifts.

#### 2.1.1. Slice Mapping With Dynamic Programming

The best cutting angle is the angle that maximizes the similarity between all histological slices and their corresponding best matching slices in the atlas. Because in-plane rotation can be fixed, we only consider rotation angle α about the superior-interior (y) axis and β about the left-right (x) axis. To solve the problem, we first find the best matching slice for each experimental slice given a potential cutting angle. The problem can be represented as follows: Let I_1…*N*_ with spacing s_*E*_ be the experimental slice sequence, and *V*_*A*_ be an isotropic atlas with voxel dimension s_*A*_, defined on the domain Ω. After rotating the atlas with potential best rotation *R*_αβ_, we reslice the rotated atlas into coronal slices and re-index them as atlas slice sequence J_1…*M*_. Using the L2 norm of HOG differences described in Section 2, we aim to find a mapping that matches each slice in I to a slice in J which minimizes the overall difference.

Taking into account potential compression along the longitudinal axis, slice quality variation, and intersubject variation, we formulate the problem with a single subset A of all slices I, where A is an ordered selection of 1…*N*, which may or may not be the whole sequence of experimental slices (depending on the image sequence quality and the value of s_A_ and s_E_). A is chosen to span the full sequence while avoiding damaged slices.

We formulate this slice mapping and difference minimization problem as a dynamic program. Let I_*A*_ be the ordered selection of experimental slices, and let J be the resliced atlas sequence ordered from the same direction along the longitudinal axis and spacing s_*A*_. The cost, *C*(*i, j*), is defined as the minimum cost of mapping the first i slices in A to a sequence of j slices, where the i^th^ slice has to be mapped to the *j*^th^ slice:

(1) $$C(i,j)=\left\{ \begin{array}{l} \;\rho (I_{A_{i}},J_{j}),\text{ if }i=1\\ \min_{k}(C(i-1,k)+\rho (I_{A_{i}},J_{j})),\text{ else} \end{array}\right.$$

where *i* ∈ *A*, 0 ≤ *j* ≤ **card**(*J*), ρ(*a, b*) denotes the difference score between Slice a and Slice b measured with the HOG similarity metric. To reduce the required computation, we only look at potential matching slices, k, which have plausible spacing:

(2) $$\begin{array}{r} |\frac{s_{\text{A}}\left(j-k\right)}{s_{\text{E}}\left(A_{\text{i}}-A_{\text{i}-1}\right)}-1|<\theta \end{array}$$

where *A*_*i*_ is the original index of the ith slice in the selected sequence and *θ* is a user-defined threshold value. This spatial constraint constrains the ratio of the distance between slices in the atlas and the experimental slices match to *θ*.

We denote the best *k* that satisfies Equation 2 and is used to fill in the cost matrix (Equation 1) as k^*^. The best intermediate steps are saved by updating the three-dimensional array M for each *i, j*:

(3) $${ℳ}_{\alpha \beta }\left(i,j\right)=\{\begin{array}{l} \begin{array}{ccc} \;\left[\begin{array}{c} j \end{array}\right], & \text{if} & i=1\\ \left[\begin{array}{cc} {ℳ}_{\alpha \beta }\left(i-1,k^{*}\right) & j \end{array}\right], & \text{else} & \; \end{array} \end{array}$$

M_*αβ*_(*i, j*) lists the the indices in J that best match each of the first i slices in A, where Slice i in A is mapped to Slice j in J and the atlas is rotated with angle α about the y axis and β about the x axis. The optimal mapping is therefore given by:

(4) $$\begin{array}{r} M_{\alpha ,\beta }^{*}\left(I_{A}\right)=M_{\alpha ,\beta }\left(\text{card}\left(A\right),j^{*}\right) \end{array}$$

where

(5) $$\begin{array}{r} j^{*}=\underset{j}{\arg\min}C\left(\text{card}\left(A\right),j\right) \end{array}$$

The cost of mapping all slices in A to resectioned slices in J with atlas rotated by αβ is given by *C*(**card**(*A*), j^*^).

#### 2.1.2. Cutting Angle Difference Determination

After running this dynamic program with different sectioning angles we should be able to directly choose the angle that gives minimum cost score to be the best cutting angle. However, since HOG is relatively insensitive to local distortions and each slice is slightly distorted, when summing up all the costs we also sum up a lot of noise. Therefore when the angle is very close to the true sectioning angle, the difference among neighboring angles is not substantial. To improve our robustness, we use a different approach. This approach also predicts how we should adjust the rotation and prevents exhaustive searching in the previous approach.

Biological structures change quickly along the posterior-anterior direction. It is not hard to tell if an experimental brain is sectioned with a different angle than the atlas volume, even if the angle deviation is only several degrees, because structures that appear in the same slice in the atlas will be in different slices in the experimental slice sequence. For example, if the left side of the brain is tilted to be more anterior, on average the right hand half coronal brain slice will appear to be more posterior to the left half. Thus if we match the left and right half slices of an experimental brain separately to the atlas, we will see that the slice number of the matching slices of the left half brain will be on average higher than that of the right half. Based on this idea, we use matching slice index differences of half brains to determine if a rotation angle best fixes the cutting angle difference between the experimental brain and the atlas.

**Figure 3:**
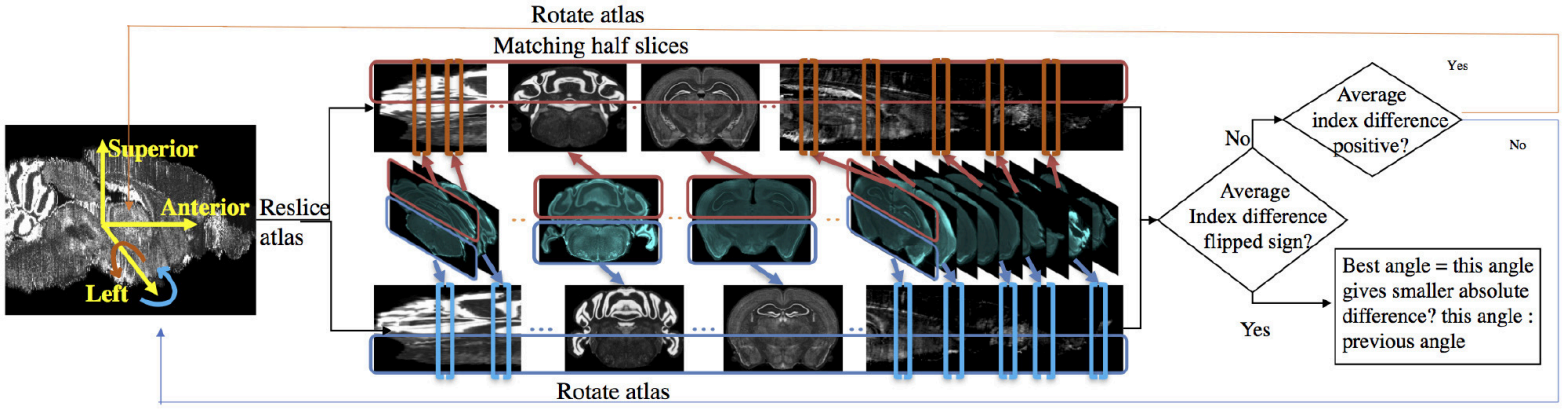
Flow chart for determining sectioning angle about the left-right (x) axis. In the matching half slices step, atlas is stretched for better illustration.

Because mouse brains have left-right symmetry, the rotation angle α about the superior-interior (y) axis tends to be around zero. The rotation angle β about the left-right (x) axis tends to be larger because the mouse brain is not flat at the bottom and can easily be set tilted on the microtome plate. Here we use the determination of angle β as an example; the flowchart is shown in Figure 3.

To find the best rotation angle β about the x axis, we solve the slice mapping problem with the method described in Section 2.1.1 on the upper half slices and the lower half slices respectively. We take the index difference between the optimal mapping given by:

(6) $$\begin{array}{l} D=\\ \frac{1}{card(A)}\sum \left(M_{\beta }^{*}(M_{upper}(I_{A}))-M_{\beta }^{*}(M_{lower}(I_{A}))\right) \end{array}$$

where M_*upper*_ and M_*lower*_ denote binary masks to apply to both experimental image and resliced atlas image to include only half of a slice. Positive *D* means upper half experimental slices are matched to slices more anterior than lower half slices. Therefore the atlas should be rotated more in the positive direction about the left-right (x) axis, where the positive direction is defined by the right-hand rule around the x axis. If *D* is negative, then the atlas should be rotated in the negative direction. We reslice the atlas again after rotation and repeat the above steps until the index difference flips signs meaning we need to rotate the atlas in another direction. The rotating angle changes in a step size of one degree. When the flipping of sign occurs, we choose the angle between the current angle or the previous angle which gives the smaller absolute index difference. The same steps are repeated to determine α.

After finding the optimal rotation $R_{\alpha \beta }^{*}$, we apply the mapping method on full slices in A to find their corresponding full slices in the optimally rotated atlas. We then interpolate linearly on the matching slice indices to find the best matching slice for every other experimental slice in the experimental volume that is not selected in A.

### 2.2. Coherency-Based 2D Deformable Registration

After the 2D to 3D registration, we map all the experimental slices to their computed corresponding slices in the optimally rotated atlas with the deformable registration to build a pixel-wise mapping from the 2D slice sequence to the atlas volume. Let an experimental image g that is globally transformed to the same coordinates of its corresponding slice be the target image, and its best matching slice f be the source image, where Ω⊂ℤ^2^ is the image domain. In the task of 2D registration, we aim at finding a transformation T such that

(7) $$\begin{array}{r} g\left(x\right)=f\left(T\left(x\right)\right),\forall x\subset \Omega \end{array}$$

where g and f become equivalent in terms of anatomical structures.

For registering histological images, the most common approach has been mutual information based free form deformation (FFD) (Rueckert et al., 1999). Like in FFD, we superimpose a uniformly-spaced sparse grid G ⊂ Ω. Because of the properties of the experimental images described in the previous sections, we continue to use the HOG difference as the similarity metric but with a smaller HOG cell size to fix local distortions. Because HOG is not differentiable, we build our work on a discrete Markov Random Field (MRF) approach (Glocker et al., 2008), where for each node *p* ∈ G we seek to assign a label *l*_*p*_ ∈ *L* that minimizes an energy function *E* consisting of two unary terms that ensures similarity to the corresponding atlas slice and to the previous experimental slice—HOG difference is used as the similarity metric—and a pairwise term that regularizes motion between neighboring nodes. The similarity-to-its-previous-slice term is added to ensure that same features in experimental slices are aligned, because some features that exist in our experimental datasets do not always have corresponding features in the atlas. Each label *l*_*p*_ corresponds to a displacement *d*_*p*_ in a predefined displacement set D_*p*_. We define the bijective function *b*_*p*_ between *L*_*p*_ and D_*p*_ for each node *p* as *b*_*p*_: *d*_*p*_ → *l*_*p*_.

#### 2.2.1. Model Elasticity With the Pairwise Term

The ventricular system spans throughout the brain, providing fluid pathways in the brain and creating regions of empty space in almost every histological slice. Those cavities are easily deformed during slice preparation procedures and have much inter-subject variation. Thus when computing this MRF warp field, one needs to take into account the elasticity of different regions in the brain. By warping images to match with each other, we are essentially warping tissues: the more two adjacent control points are displaced, the more tension accumulates, if the two control points are connected through coherent tissue. In contrast, if they are separated by any hollow structure or empty space, no tension should be built in between.

The traditional and most common interpolation method for biomedical image analysis has been the B-spline model (Rueckert et al., 1999), where each pixel is affected by 4 × 4 neighboring control points. In the case where two control points are separated by an empty space, a B-spline interpolation no longer makes sense because of discontinued tissue coherency. Therefore to better model tissue deformation, we use the simple bilinear model where a point is only affected by its direct 2 × 2 neighboring control points. Of course, now our warp generation needs to ensure some smoothness.

**Figure 4:**
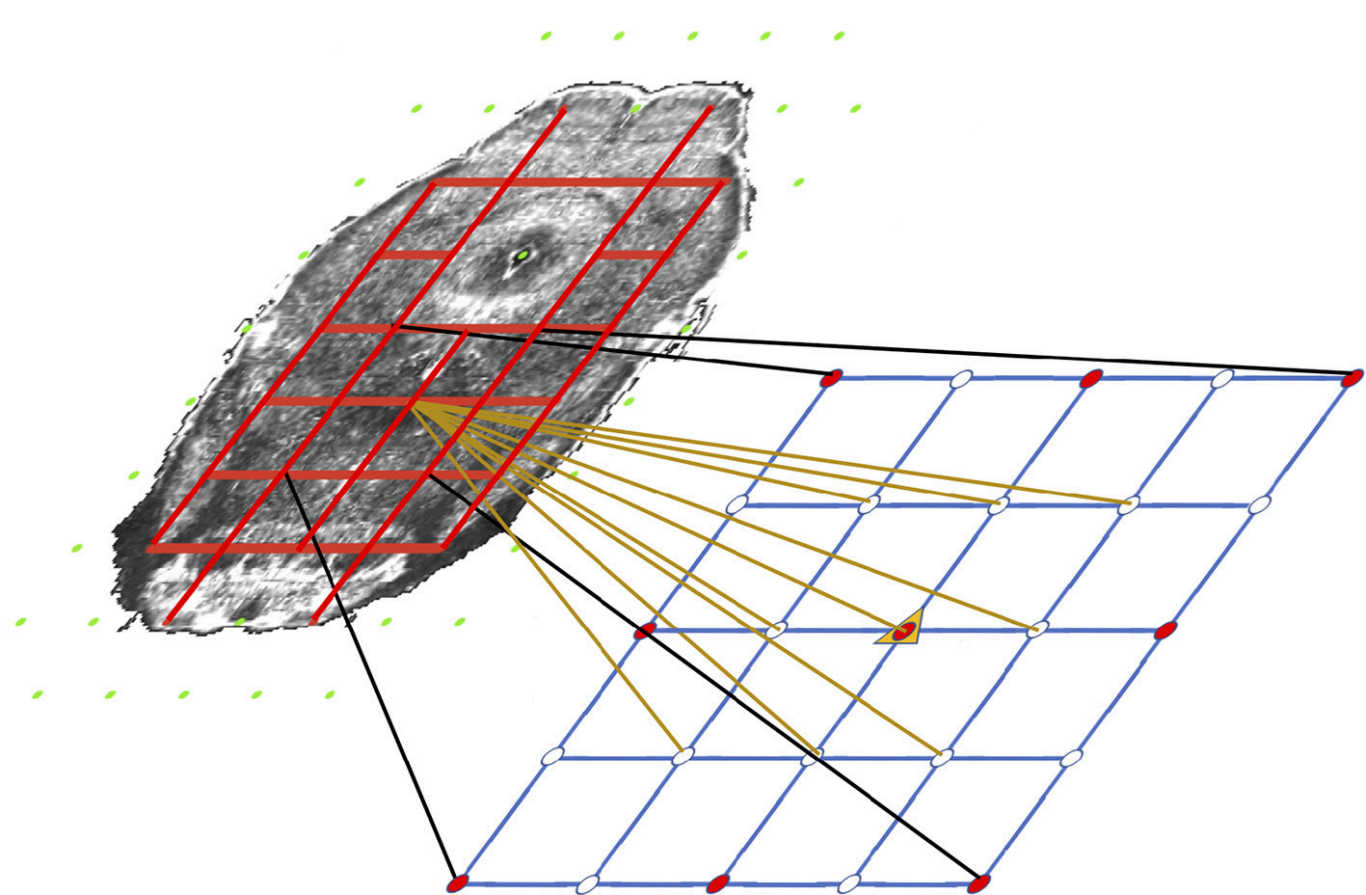
Illustration of the coherency model and grid refinement from level t to level t+1. Grids are overlaid on an atlas image (contrast adjusted for better illustration) to show the coherency model. Green nodes are free nodes which include nodes in the free regions—ventricle systems and background—and affect real tissue. Red nodes are coherent nodes which cannot be seen since they become part of the tension edges, represented by red line segments between coherent nodes. During refinement, image resolution is 2 × the resolution of last level. The grid spacing remains the same. Therefore the grid quadruples in each direction, which is shown in the lower grid. The motion of existing nodes are carried onto the next level. The motion of non-existing nodes in the lower grids are interpolated from the motion of the existing nodes.

Our system does so with a very simplistic model. We divide each target slice into two regions: free (ventricular system and background), and coherent (other areas) based on the annotation volume of ABA. We then classify the nodes as coherent (red) or free (green in Figure 4) based on if they are inside a coherent or a free region. The idea is tension only accumulates when we compress or stretch two nodes that are connected solely with a coherent region. If there is an empty space between two nodes, intuitively compressing them or stretching them should not build tension in between. Based on this property, we use the pairwise term—the traditional regularization term—to model tension accumulated between nodes with which we stretch or compress brain tissues. There is an edge (red line segment in Figure 4) only if the connecting line segment between the two nodes only crosses coherent regions which indicates both nodes must be coherent as well.

We first extract mask images *r*_*c*_ and *r*_*e*_ representing coherent region—tissue—and empty space region— ventricles and background—respectively from the annotation volume of the ABA and project them to the source image. We further group control points as coherent or free in Equation 8. Coherent control points are inside coherent regions. Free control points are the control points inside an empty space, and moving the control point will affect pixels inside coherent regions.

(8) $$p\in \{\begin{array}{ll} G_{\text{ coherent}}, & \text{ if }r_{c}(p)=1\\ G_{\text{free}} & \text{ if }\sum_{x\in \Omega }\left[\eta^{-1}(|x-p|)\cdot r_{c}\right]>0\\ \; & \text{ and }r_{e}(p)=1| \end{array}$$

where the inverse influence function, η(.)^−1^, adopted in Glocker et al. (2008), masks pixels influenced by a control point p. We include the influence function in control points classification, because we only care about control points that affect image appearance.

We further define a tension edge set, **E**, where tension accumulates when moving the two control points connected by an edge in this set. Basically an edge *e*_*pq*_ is in **E** if the line segment connecting Node *p* and Node *q* only crosses coherent region *r*_*c*_. Because the spring potential energy is proportional to the square of displacement, we use squared difference as the pairwise term:

(9) $$\begin{array}{r} V_{pq}\left(l_{p},l_{q}\right)=\lambda {\left(d_{l_{p}}-d_{l_{q}}\right)}^{2},e_{pq}\in \text{E} \end{array}$$

where λ is a regulation parameter.

#### 2.2.2. Multi-Level Estimation

We need to be able to both correct large distortions and make small changes to achieve good results. For both computation efficiency and quality of results, we use a multilevel approach. Since we are trying to model the tension that the deformations create, we need the pairwise energy terms to accumulate as we refine the grids. This requirement means that we cannot use the approach used by Glocker et al. (2008), but instead solve the problem using a method where each refinement level maintains knowledge of the distortions created by previous levels.

The conventional multilevel approach (Glocker et al., 2008) repeats the same procedure with progressively finer grids: locations of the control grid points that minimize the unary and pairwise terms are computed, and then the resulting image is warped to match these new grid locations. The next level grid is added to the warped image, and the process is repeated. To maintain tension in a realistic way, we do not reset the grids and tension after each iteration and use each iteration to simply update the allowable possible positions (labels) for the next iteration. More formally, to carry the squared form tension correctly to the next level, at every level *t* for each node *p* we update the possible discrete displacements $M_{t}^{p}$ to reflect the accumulated prior displacements of the node plus the current displacement to be evaluated at the current level.

To correctly carry on calculated displacements to the next level, we first need to compute the set of possible locations for each grid point, which depends on the results from the prior level. To do this, we denote the grid at level t as G^*t*^ and the influence function as η^*t*^. Let $d_{l_{p}}^{t}$ be displacement at node *p* with label *l* at level t. At each level t, we estimate best motion $d_{l_{p}}^{t}$ at each node *p*∈G^*t*^ and bilinearly interpolate them to get the initial displacement for each node in G^*t*+1^ at the next level. We denote this preset displacement at node *p* as ${\bar{d}}_{l_{p}}^{t+1}$, therefore the set of possible displacement at node p level t+1 is given by the sum of this preset displacement and possible displacement in level t+1:

(10) $$\begin{array}{r} D_{p}^{t+1}=\left\{d:d={\bar{d}}_{t+1}^{l_{p}}+\theta ,\theta \in \Theta \right\} \end{array}$$

where Θ is the allowable additional displacements and is the same for every node.

Having created a set of possible locations for each grid point, we next need to create the image that we will compare at this level to compute the similarity. In previous work, this warped image is input to this level, but we need to compute the image from the displacements of the previous level's control points and the labels associated with the node we are evaluating. When estimating the local patch around node *p* at level *t*+1, we use the positions of the grid points that are around p from the prior level and the position of *p* for the given label at this level. This provides an estimate that incorporates the warp from the prior level and an estimate of the additional warp created by moving p to the position indicated by this label at the current level. For simplicity and computational efficiency, this estimate ignores the warp that will be caused when other control points at this level move.

We denote the patch that is affected by p in the first level function $\eta_{0}^{-1}\left(\left|x-p\right|\right)$ as R_0, *p*_. The control points in the patch at level t+1 is defined as:

(11) $$\begin{array}{r} N_{p}^{t+1}=q\in G^{t+1}:\eta_{0}\left(\left|q-p\right|\right)>0 \end{array}$$

To create the image that we will compare, we set the nodes in N_*p*_ at the values from level *t*, except for node *p* which we evaluate with the displacements from the current level in the set $D_{p}^{t+1}$. Therefore the transformation applied to the affected region R_0, *p*_ when we associate label *l*_*p*_ with node *p* at level *t* + 1 is:

(12) $$\begin{array}{r} T_{p}^{t+1}\left(x,l^{p}\right)=x+\underset{q\in N_{p}^{t+1},q\neq p}{\sum }\eta \left(\left|x-q\right|\right)d_{l_{q}}\\ +\eta \left(\left|x-p\right|\right)d_{l_{p}} \end{array}$$

Thus the unary term is given by the similarity measure between the warped patch and target patch:

(13) $$\begin{array}{r} V_{p}^{t+1}\left(l_{p}\right)=\rho \left(g\left(x\right),f\left(T_{p}^{t+1}\left(x,l_{p}\right)\right)\right),l^{p}\in D_{p}^{t+1} \end{array}$$

where *x*∈R_0, *p*_ and ρ measures the difference score between two images. Since at every level, the only region that changes when we approximate the change for each possible label is centered around the node being evaluated, we approximate this change by simply translating the patch centered at the approximate new node coordinates $p+d^{l_{p}^{t}}$ with possible additive displacements $d^{l_{p}^{t+1}}$ at the current level and compare the HOG similarity with the patch that is centered at the node's original coordinates in the fixed image or the previous image. Therefore the unary term is estimated as:

(14) $${\bar{V}}_{p}^{t+1}(l_{p})=\rho (g(R_{t,p}),f(R_{t,p+d^{l_{p}^{t}}+d^{l_{p}^{t+1}}}))$$

where R_*t, p*_ denotes the patch centered at node *p* in the fixed image, and $R_{t,p+d^{l_{p}^{t}}+d^{l_{p}^{t+1}}}$ denotes the patch centered at newly estimated coordinates of displaced node *p*.

Eventually we formulate the MRF energy function at level t as the summation of the normalized unary similarity term to the corresponding atlas image, the unary similarity term to the warped previous image, and the pairwise term:

(15) $$\begin{array}{ll} E^{t} & =\sum_{p\in G^{t}}F\circ V_{p,atlas}^{t}(l_{p})+\sum_{p\in G^{t}}F\circ V_{p,prev}^{t}(l_{p})\\ & +\sum_{p\in G^{t}}\sum_{q\in \{q:e_{pq}\in E^{t}\}}F\circ \;V_{pq}^{t}(l_{p},l_{q}) \end{array}$$

where F denotes the normalization operation. We obtain the HOG difference for all nodes and all labels at each level and normalize the matrix so that the values are within range [0, 1]. The pairwise term is normalized in the same fashion.

Because the free nodes are not constrained with any pairwise terms, they are essentially assigned labels that minimize the unary terms:

(16) $$l_{p}^{t}=\underset{l\in b(D_{p}^{t})}{\arg\;\min}V_{p,atlas}^{t}(l_{p})+V_{p,prev}^{t}(l_{p})$$

where $p\in G_{free}^{t}$. Coherent labels are solved by minimizing the energy function:

(17) $$\begin{array}{l} \;E_{coherent}^{t}=\sum_{p\in G_{coherent}^{t}}F{}^{\circ}V_{p,atlas}^{t}(l_{p})+\\ \sum_{p\in G_{coherent}^{t}}F{}^{\circ}V_{p,prev}^{t}(l_{p})+\sum_{p\in G_{coherent}^{t}}\sum_{q\in \{q:e_{pq}\in E^{t}\}}F{}^{\circ}V_{pq}^{t}(l_{p},l_{q}) \end{array}$$

#### 2.2.3. Contour Alignment With Symmetric Difference

**Figure 5:**
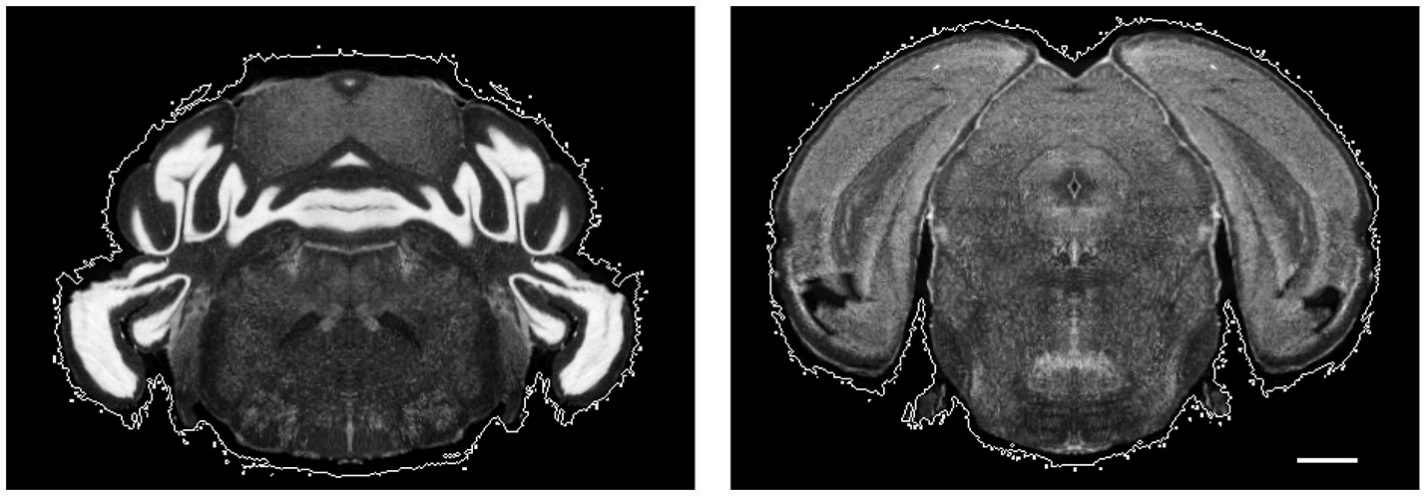
Example atlas slices with nonzero intensity regions circled by white contours. Scale, 1 mm.

While HOG matches internal features well, we find it hard to align the contour of images. The atlas has very low-intensity pixels around real brain tissues as shown in Figure 5. Because HOG's relative insensitivity to intensity, this noise can cause errors in contour alignment. We added an intensity threshold in the process of computing HOG—if a pixel's intensity is lower than the threshold, its gradient is not included in the histogram. However, this does not solve the problem, because a single threshold intensity cannot eliminate the background noise perfectly. To remove the unwanted background, we make use of the fact that the corresponding slice's annotation in the annotation volume of ABA is conservative: it is inside the actual imaged tissue. Thus all pixels inside the annotation are foreground pixels. We further segment the pixels that are annotated as background in the annotation to the foreground and real background with the intensity information of the image. We build an energy function so that the unary terms try to minimize intensity difference among the pixels that are in the same class, and the pairwise term encourages two neighboring pixels to be grouped in different classes when the intensity difference is large and to be grouped in the same class when the intensity difference is small:

(18) $$\begin{array}{l} E(f)=\sum_{p\in V}1(f_{p}=0)\cdot i_{p}+\sum_{p\in V}1(f_{p}=1)\cdot |i_{p}-I_{average}|\\ \;+\sum_{p,q\in ℰ}e^{-{\left(i_{p}-i_{q}\right)}^{2}} \end{array}$$

where V represents all the pixels to be classified - the pixels are annotated as background in the annotation, E denotes pixels that are in the 4-connected neighborhood, f denotes the assignment of background - 0 or foreground - 1, *i*_*p*_ represents the intensity at pixel *p*, and *I*_*average*_ is the average intensity of all nonzero pixels in the image. Solving this energy function, we can obtain satisfactory result except that some very dark tissue regions near a slice's contour will be removed in some slices. To fix it, we keep the otherwise removed regions if the area is well-connected with its surrounding regions. This is accomplished by morphological eroding and dilating the to-be-removed regions with a disk of 20 pixels. We keep a region if it survives the opening operation. Same numbers are used across slices. We fill in holes in the computed mask so that the mask consists of a single piece. Even though the experimental slices are often preprocessed by neuroscientists to remove the nonzero-intensity background and keep only the tissues, this procedure is not quality-controlled. Therefore we refine the experimental images again with a similar method that is used to preprocess the atlas images, except that the masks are morphologically eroded and dilated with a disk of 3 pixels to encourage smoothness, and the to-be-removed regions will be kept if its area is greater than 50 pixels. These parameters were selected based on experiments on several slices in one of our experimental brains. Same parameters are used for all experimental slices. In the case that a slice is missing a relatively large portion of tissue, after the plane correspondence is found and before the 2D nonrigid registration, a manual preprocessing is done on the corresponding atlas slice to crop out the same corresponding portion that is missing in the corresponding atlas plane returned by our algorithm.

After fixing the background noise, we find it still hard to use HOG difference to align image contours because HOG difference reduces dramatically only when after transformation the contours overlap or are separated by a distance smaller than the HOG cell size. Moreover, since the atlas is not a smooth volume, after rotation, the contours may be jagged - creating unwanted gradients. A more sensitive and more robust metric is needed. When displacing nodes that affect image contours, we are essentially warping the contour to maximize overlapped region of the two images or equivalently to minimize the symmetric difference of image foregrounds. Therefore, if a node *p* influences contour pixels, we modify the HOG difference term to its corresponding atlas patch to be the symmetric difference of the two tissue masks. Because node *p* is essentially a free node, Equation 17 is updated to:

(19) $$\begin{array}{l} l_{p}^{t}=\underset{l\in b(D_{p}^{t})}{\arg\;\min}\sum_{\eta^{-1}(|x-p|)=1}(m_{f}(x)-m_{g}(x))+V_{p,prev}^{t}(l_{p})\text{ if}\\ \;\sum_{x\in \Omega }\eta^{-1}(|x-p|)\cdot c_{e}>0 \end{array}$$

where the contour of the experiment image's real tissue is denoted by *c*_*e*_, and the real tissue in image *f* and *g* are *m*_*f*_ and *m*_*g*_. In Equation 17 we estimate the warped patch by translating the patch center. This estimation improves computation speed while retaining performance when the similarity measure is HOG difference or another metric that involves more internal information. The shape of the experimental images is often deformed in the preparation process. However, simple translation does not change the shape. It only reduces the disjoint area but cannot find a transformation that reverts the deformation. Therefore instead of simply translating the patch, we warp the binary masks to evaluate this symmetric difference term.

### 2.3. Improvement Based on Data-Specific Properties

**Figure 6:**
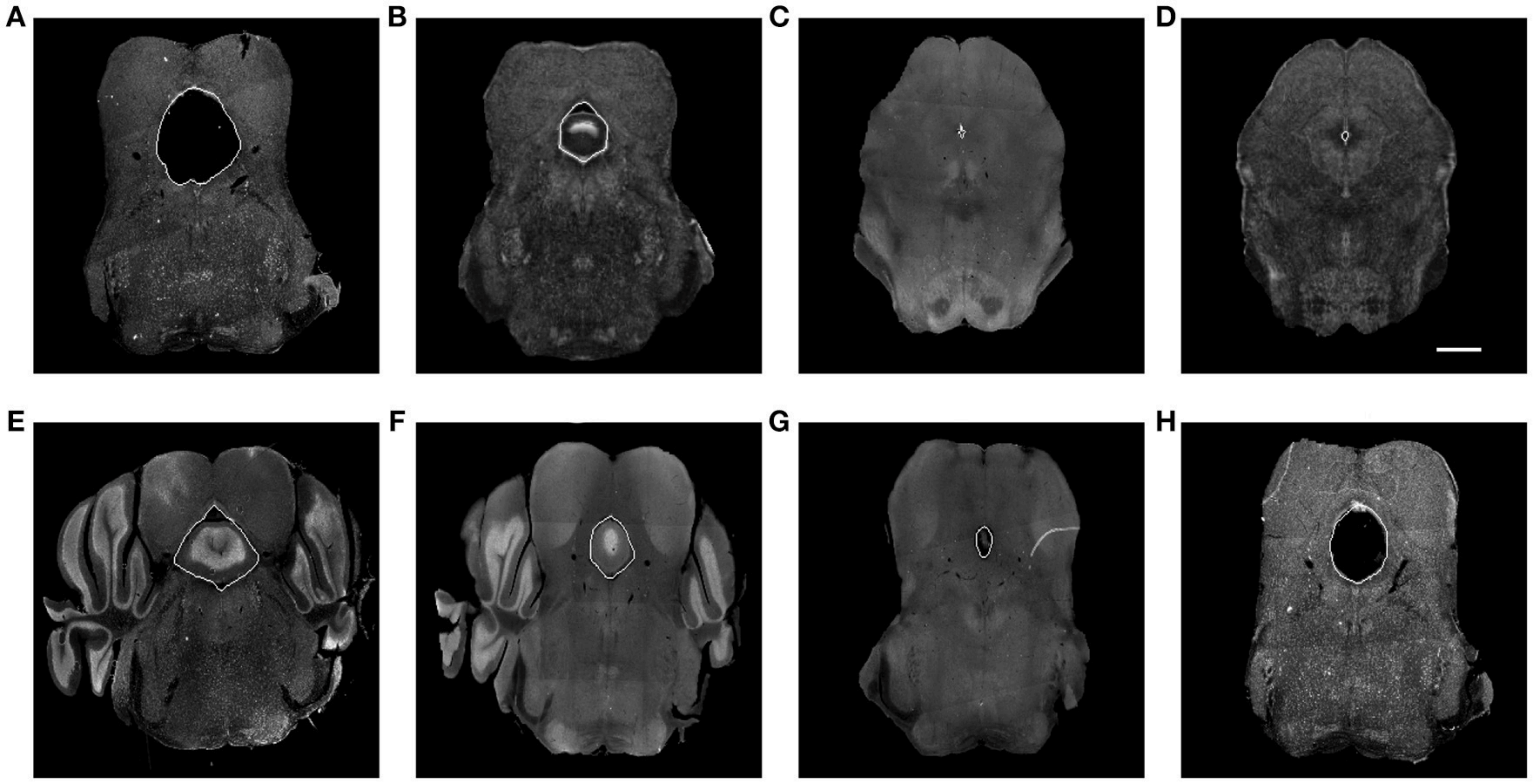
**(A)** Expanded aqueduct in an experimental image. **(B)** Aqueduct in the corresponding atlas image of **(A)**. **(C)** Squeezed aqueduct in an experimental image. **(D)** Aqueduct in the corresponding atlas image of **(C)**. **(E–H)** Various aqueduct appearance in different brains and slices. Aqueduct contours are marked with white curve. Scale, 1 mm.

Our framework was used in a systematic anatomical study in the hindbrain to map the brain regions containing the dorsal raphe nuclei to the ABA to study the organization of the dorsal raphe serotonin system and its behavioral functions related to depression and anxiety (Ren et al., 2018). The dorsal raphe nuclei are ventral to a hollow structure called aqueduct. Due to the difference in brain preparation procedure, for example, the dehydration step, as illustrated in Figure 6, the aqueduct shows variability between our experimental brains and the atlas. The difference in size, appearance, and edge orientation of the aqueduct makes aligning the regions around it difficult using the coarser grained HOG descriptor alone. This situation is made even more difficult because a squeezed aqueduct can be smaller than the grid size in the finest iteration.

We solve the squeezed aqueduct problem by warping the segmented aqueduct to the corresponding annotated atlas aqueduct with thin plate spline (TPS) (Bookstein, 1989). Because aqueduct appearance varies across subjects, sectioning angles, and longitudinal axis as shown in Figure 6, it is hard to segment them with a single traditional segmentation method. We trained manually labeled aqueduct with a network similar to Chen et al. (2017) implementation of context aggregation (Yu and Koltun, 2015), a convolutional network designed for dense prediction. To build point correspondence on the aqueduct contours, we find the highest and lowest point on the aqueduct contours. If there are multiple highest or lowest points, we choose the point that is closer to the centroid of the aqueduct contour in the horizontal direction. We divide the contour into halves with the highest and lowest points and build point correspondence by uniformly sampling the same number of points along the curve. The points on the aqueduct contours only ensure the alignment of inside the aqueducts. Since the images are mostly aligned with MRF, we include the control points outside of both aqueducts to add more control to the TPS warp.

With point correspondence on the aqueducts' contours, we add another term to the unary term so that the aqueduct is brought closer before reshaped with TPS. The term measures the Euclidean distance between the warped experimental aqueduct contour points and their corresponding atlas aqueduct contour points:

(20) $$\begin{array}{r} D_{p}^{t}\left(l_{p}\right)=d\left({\text{v}}_{\text{p}},T_{p}^{t}\left({\text{u}}_{\text{p}},\text{p}\right)\right) \end{array}$$

where an experimental aqueduct contour point *u* ∈ **u**_**p**_, if its influence to node p - η_*t*+1_(|*u* − *p*|) > 0. **v**_**p**_ are the corresponding aqueduct contour points in the atlas image, and *d* measures the Euclidean distance between two sets of points. Therefore the energy function for coherent nodes in Equation 18 becomes:

(21) $$\begin{array}{r} \begin{array}{c} E_{coherent}^{t}=c_{a}\underset{p\in G_{coherent}^{t}}{\sum }F{}^{\circ}V_{p,atlas}^{t}\left(l_{p}\right)+c_{b}\underset{p\in G_{coherent}^{t}}{\sum }F{}^{\circ}V_{p,prev}^{t}\left(l_{p}\right)+\\ c_{d}\underset{p\in G_{coherent}^{t}}{\sum }F{}^{\circ}D_{p}^{t}\left(l_{p}\right)+c_{p}\underset{p\in G_{coherent}^{t}}{\sum }\underset{q\in \left\{q:e_{pq}\in {\text{E}}^{t}\right\}}{\sum }F{}^{\circ}V_{pq}^{t}\left(l_{p},l_{q}\right) \end{array} \end{array}$$

where *c*_*a*_, *c*_*b*_, *c*_*d*_ and *c*_*p*_ are the coefficients before the energy terms. We assign labels that minimize the new combined unary term to the free nodes:

(22) $$l_{p}^{t}=\underset{l\in b(D_{p}^{t})}{\arg\;\min}c_{a}V_{p,atlas}^{t}(l_{p})+c_{b}V_{p,prev}^{t}(l_{p})+c_{d}ℱ\circ D_{p}^{t}(l_{p}))$$

where $p\in G_{free}^{t}$.

### 2.4. Implementation

#### 2.4.1. HOG Cell Size

We use a cell size of 15 pixels to measure the image similarity (see Section 3.1.1). This relatively large cell size allows us to capture structural similarities even with uncorrected small distortions. For nonrigid registration in Section 2.2, we decrease the cell size to 4 pixels, because the purpose of this step is to correct distortions. In both steps, the block size is 2 × 2. HOG is computed with the Vlfeat toolbox (Vedaldi and Fulkerson, 2010).

#### 2.4.2. Set A

We select a subset A from all the slices I to find the best cutting angle and the best corresponding slices. For full brain data, which contains about 200 slices, we use about 30 slices with minimal artifacts - 1/6 to 1/7 of the whole sequence. In the anatomical study, each sectional brain consists of about 35 slices. We use every third image for most of the brains - about 12 slices for each experimental brain. For some brains with relatively more damaged slices, we manually checked the automatic selection and replaced slices with significant damage with a nearby good quality slice. Since many slices are used to find the best cutting angle, this manual check and replacement is only performed when many automatically selected slices are damaged. With Matlab, it takes 38.8s on a 12-core 3GHz Linux machine to evaluate a set of 12 slices, or equivalently to evaluate a cutting angle on a sectioned brain.

#### 2.4.3. Parameter Selection in the Energy Function

All terms—the unary terms and the pairwise term—in the energy functions presented in this paper are normalized to the range [0, 1]. Since the experimental images and the atlas volume are of the same modality, and the terms are normalized, the parameters before each term do not need to be heavily tuned to yield good results. Our original energy function consists of only one unary term—the HOG similarity term to the atlas slice—and the pairwise term. With several experiments, we find equal weight between the unary and the pairwise term generates the best visual result. In the general energy function in Equation 16, the HOG similarity term to the previous slice is added to encourage smoothness in the “reconstructed” volume and make the features that do not exist in the atlas but exist in the experimental volumes more consistent. We add an additional Euclidean distance term between the two aqueduct contour point sets in Equation 22 to suit our dataset better. Since the HOG difference to the corresponding atlas slice is the dominant term, we set it to be three times as strong to the HOG difference term to the previous slice and the Euclidean distance term between the two aqueduct contour point sets in both forms of the energy function. The coefficients before the pairwise term is set to be the sum of the previous coefficients to maintain the equal weight between unary and pairwise terms.

#### 2.4.4. Iteration Details

We use three iterations to complete the 2D nonrigid registration described in Section 2.2. The grid spacing is 16 × 16 in all iterations. In the first iteration, we downsample both images 4 × in both horizontal and vertical directions. In the second iteration, images are downsampled by 2 × . In the final iteration, we use the original resolution. The maximum displacement at each level is set to be half of the grid spacing. Therefore the total number of labels are 17 × 17 in each iteration. The optimization is computed with tree-reweighted message passing (TRW), more specifically TRW-S (Kolmogorov, 2006; Chen and Koltun, 2015).

#### 2.4.5. Segmentation

We chose five brains from all our brains that could represent the variability of aqueduct appearance and label the aqueduct of all slices in the selected brains. Both the experimental slices and aqueduct masks were downsampled to 512 × 512. One brain in these five brains happens to be in the five brains that we evaluate in the Evaluation Section 3.2. Because our training data consists of only five brains-about 150 slices, we data-augmented the training data and predicted the aqueduct of all other brains with this trained model. The quality of the prediction is correlated with the quality of the dataset. The generated masks are manually corrected if necessary which is about 10% of the total number of slices. The segmentation network consists of 9 layers. The input and output image has dimensions 512 × 512 × 1 where the input is the image to be segmented, and the output image is the predicted mask of the aqueduct. The first seven layers have dimension 512 × 512 × 32 with dilation rate doubling the rate of the previous layer starting from 1. The convolution kernel size is 3 × 3. The largest receptive field in the network is the seventh layer - 256 × 256. The last two layers consist of an undilated smooth layer with the same kernel size and a linear transformation layer. We use the intersection over union as the loss function and take 8 points on each half of the segmented aqueduct to compute the point distance term in the revised similarity term function in Equation 22.

## 3. Results

Our framework was developed to register a full mouse brain slice sequence consisting of 202 60 μm-thick slices to the atlas and was also used in a systematic anatomical study in the hindbrain to study the organization of the dorsal raphe serotonin system (Ren et al., 2018). We mapped all sections of the dorsal raphe region in 36 brains (hereafter referred to as “sectional brain”) in this study (Ren et al., 2018) to atlas volume of the ABA with our framework. The dorsal raphe region from each brain consists of 30 to 55 coronal slices with 40 μm to 50 μm thickness and 5.1 μm per pixel. Image size varies across brains with resolution ranging from 1 megapixels (1000 × 1000) to 6 megapixels (2000 × 3000) in the sectioning plane. Because the posterior cerebral cortex in brain sections that contain the dorsal raphe is easily detached during the slice preparation procedures, if the cortex is mostly missing in an experimental brain, we would preprocess the experimental slices manually to remove all the cerebral cortex tissues before feeding the data to our framework. The atlas volume used in this project is 320 × 456 in the coronal plane and consists of 528 slices, with an isotropic 25 μm resolution. For the brain alignment in this systematic anatomical study, we use the brain section in that atlas that contains the region of interest. The cerebral cortex in the atlas is removed if the cerebral cortex in the target experimental brain is mostly missing.

### 3.1. Evaluation Method

We use 5 brains in the anatomical study (Ren et al., 2018) to quantitatively analyze the performance of our framework. In addition, because of the lack of ground truth in these brains, we generated a simulated hindbrain and a full simulated brain from the atlas with known transformations. The hindbrain atlas section and the original full atlas are resliced with sectioning angles different from that of the original atlas. The most anterior and posterior partial slices are removed. We then remove every other slice so that the thickness is similar to the thickness of our experimental dataset. For each slice in the simulated dataset, we apply a random rotation that is smaller than 2.5°clockwise or counterclockwise, and a translation smaller than 10 pixels in each direction. The image is further warped with a randomly picked nonrigid deformation computed to map an experimental slice to its corresponding atlas slice. To generate the simulated full brain, we adjusted the deformation fields by randomly replicating the deformation fields with the largest span in the horizontal and vertical direction until the deformation field covers all the tissue area of the simulated slice. The contrast of each image is adjusted so that the illumination is different from that of the original atlas slice.

The most common metric for evaluating image registration is the target registration error (TRE) measured as the Euclidean distance between landmark point coordinates in the target image mapped by a computed transformation to the source image and the corresponding landmark points in the source image. We asked one of our neuroanatomist coauthors to identify 20 sparsely-scattered landmarks in the hindbrain of the atlas which she would be confident in locating in both simulated and real experimental brains. The points are enough to cover all the significant brain areas in this study, because 1) an experimental brain has around 35 slices, 2) on a representative experimental slice, there are roughly 30 nuclei identified by its anatomical properties based on neuroscientists' historical consensus, 3) almost all the nuclei are shown on at least 5 slices. The corresponding points of these 20 points are marked by the same neuroscientist in the brains that we evaluated. In the full brain, we select 17 regions - 81 lateral ventricle, 581 triangular nucleus of septum, 286 suprachiasmatic nucleus, 338 subfornical organ, 223 arcuate hypothalamic nucleus, 830 dorsomedial nucleus of the hypothalamus, 470 subthalamic nucleus, 884 amygdalar capsule, 587 nucleus of darkschewitsch, 214 red nucleus, 931 pontine gray, 872 dorsal nucleus raphe, 642 nucleus of the trapezoid body, 574 tegmental reticular nucleus, 169 nucleus prepositus, 222 nucleus raphe obsurus, 207 area postrem (the numbers in front of region names are the region ID in the annotation volume) and generate landmarks automatically by sampling 100 points along these brain region boundaries. These regions show contrast to their neighboring regions in at least a few slices that contain them. The sampled points cover 32% of the total number of slices and 72% of the entire brain length. We then map the selected landmark points with the known transformation to obtain the ground truth.

In the sectional simulated brain, we can compute the true error of both our method and of the expert, since we have ground truth, as well as the TRE - expert and computation combined error. This information can help interpret the results in the five experimental brains, where we can only compute the TRE. For the full brain, we are only able to measure the computation error since the landmark points are generated automatically, but the information from expert error in the simulated sectional brain can help us interpret the results.

To compare our results to previous work, we use Ju's method (Ju et al., 2006) to reconstruct the brains because it is a fully automatic method that corrects distortions nonrigidly and generates a smooth reconstructed volume which facilitates 3D registration to the atlas. We use the same exact portion of the atlas to perform this comparison experiment as in our method. We first resample the experimental slices to the resolution of the atlas slices and rigidly align them from the middle slice to the two ends. Then we nonrigidly reconstruct the sections with a five-slice neighborhood through Ju's stack Aligner (Ju et al., 2006). With stack Aligner, warp functions between every pair of slices are computed. The weighted average of these warps in the 5-slice neighborhood is then applied to every slice. Finally, the reconstructed volume is then registered to the atlas with Elastix (Klein et al., 2010; Shamonin et al., 2014) and the parameter file created by Hammelrath et al. (2016). This parameter file is developed specifically for 3D mouse brain registration and performs the best among the files that we tested. With Elastix, reconstructed volumes are first rigidly aligned, then affinely aligned, and finally elastically aligned with B-spline to the atlas. Similar to our experiment, we measure computation error on the simulated brains and the TRE on the sectional simulated brain and experimental brains.

### 3.2. Evaluation Result

#### 3.2.1. Quantitative Result

**Figure 7:**
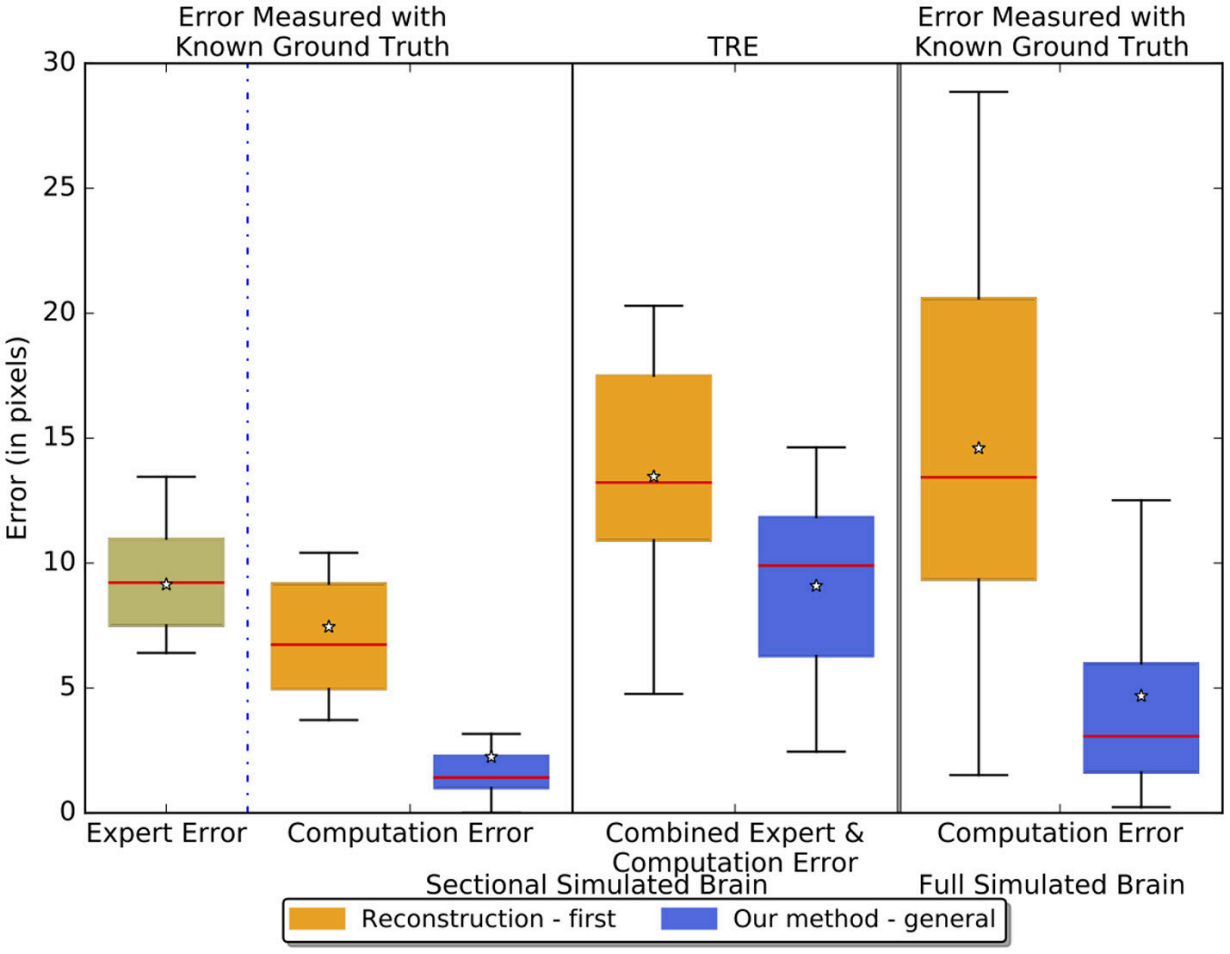
Boxplots showing the experiment results on the sectional simulated brain and the full simulated brain. The boxplots in the left two columns show the sectional brain results. We measured the intrinsic expert error, pure computation error, and the TRE - combined expert error and computation error of the reconstruction-first method and our method. The third column shows the results on the full simulated brain where we measured the pure computation error. The lines on the boxes represent the minimum, first quartile, median (red), third quartile, and maximum respectively. The star denotes the average.

Figure 7 reports the results of separately measured expert error, computation error, and the TRE - combined expert and computation error - of the reconstruction-first method and our method on the sectional simulated brain and the computation error on the full simulated brain. The expert has an intrinsic error of about 9 pixels (one pixel equals 25 μm given by the resolution of the atlas volume of the ABA) - similar to the TRE of our method on the sectional simulated brain. This figure also shows that our method, with a 2.24-pixel error on the sectional simulated brain, is about three times better than the reconstruction-first approach which has an error of 7.45 pixels. For the full simulated brain, the TRE of our method is 4.68 pixels vs. the TRE of the reconstruction-first method - 14.59 pixels. We expect the computation error to be slightly greater and show more variance for the full simulated brain than the sectional brain, because even though the landmarks are sampled on region boundaries that show contrast to neighboring regions at least in some slices, we did not further constrain on the slice numbers when we sampled the landmarks. Moreover, because the simulated deformations are taken from real experiments, when applied on a slice in the full brain, some large deformations unavoidably are applied to regions with relatively uniform intensity. Without features salient enough as in the original image where the deformation is generated, it is difficult to fully correct the deformation. The results agree with our expectations, and the error of our method is smaller than that of an expert and the reconstruction-first method.

**Figure 8:**
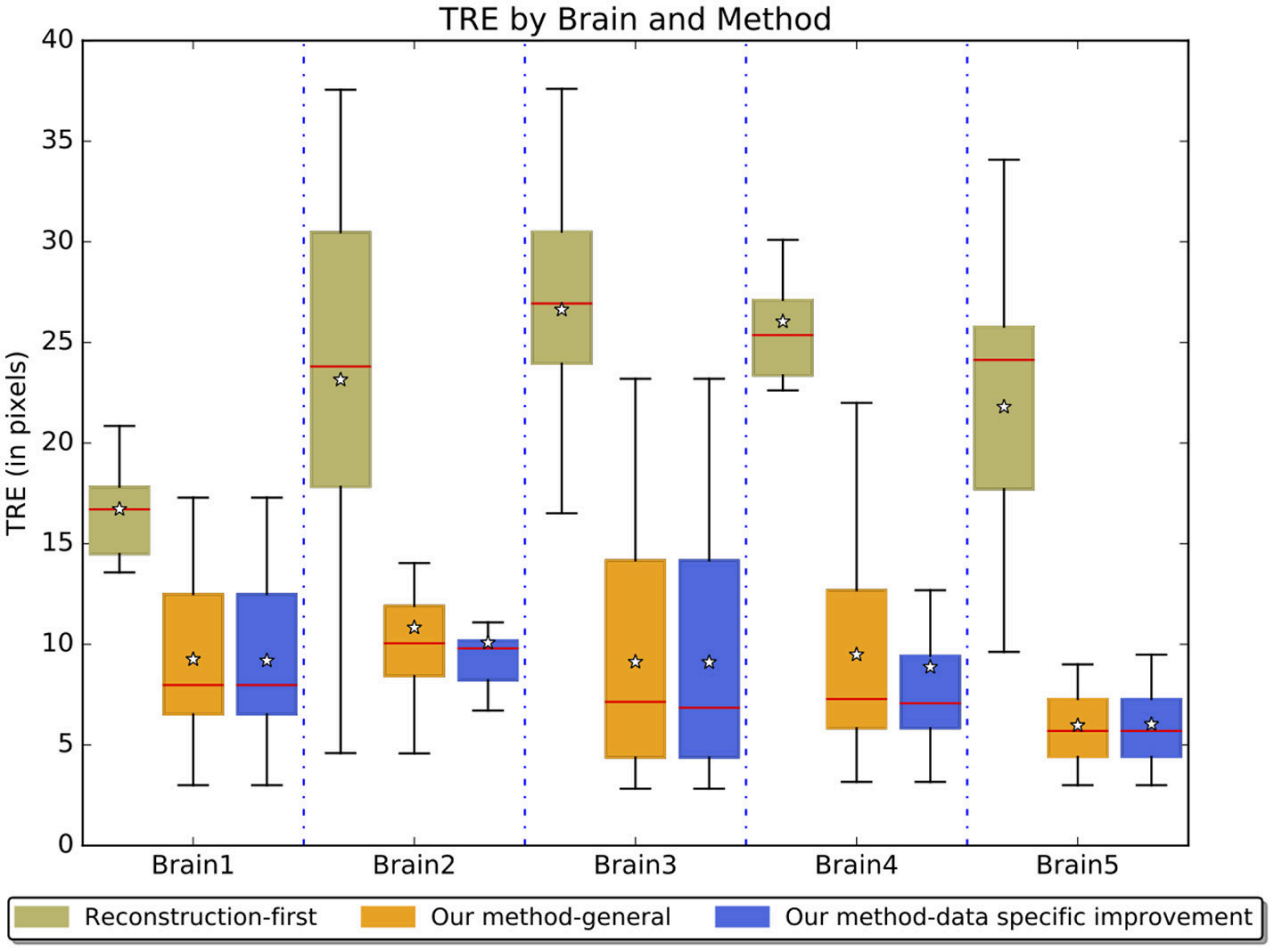
Boxplots of the TRE on evaluated experimental brains.

Figure 8 reports results on real experiments of the reconstruction-first method and our method without and with the data-specific improvement. The five brains represent some of the data variability we see in real datasets. These distances represent a combination of human and computer inaccuracy. Based on the simulated result on the sectional simulated brain, we believe the intrinsic expert error is likely to be much larger than the computation error of our method. With data-specific improvements, the average TRE slightly improves. We see a larger improvement on landmark points near the aqueduct. The TRE ratio is on average 2.56 between the reconstruction-first method and our method.

#### 3.2.2. Qualitative Result

**Figure 9:**
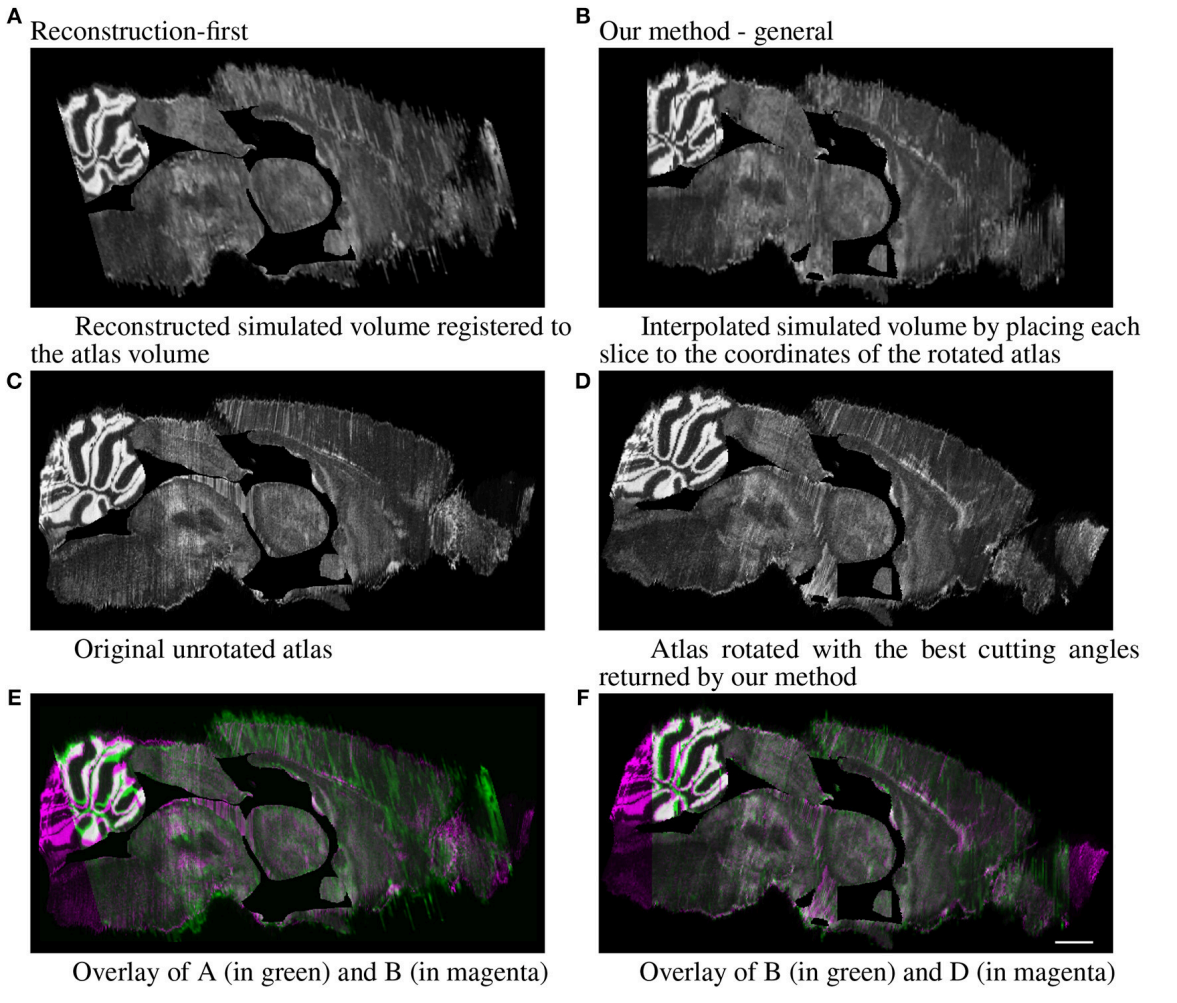
Full simulated brain results, sagittal view. Scale, 1 mm. **(A)** Reconstructed simulated volume registered to the atlas volume and **(B)** Interpolated simulated volume by placing each slice to the coordinates of the rotated atlas. **(C)** Original unrotated atlas and **(D)** Atlas rotated with the best cutting angles returned by our method. **(E)** Overlay of A (in green) and B (in magenta) and **(F)** Overlay of B (in green) and D (in magenta).

For the simulated full brain, we display the sagittal view (ventricle systems masked out) of the results generated with the reconstruction-first method and our method. The reconstruction-first method first reconstructs the brain and then aligns the reconstructed brain to the atlas. Our method approaches the problem differently by first finding the best matching angle and the corresponding slices in the resliced atlas for each experimental image, then registers each experimental slice to their corresponding slice individually. To show the “reconstructed” brain with our method, we place each slice to the coordinates of the atlas volume rotated with the best cutting angles and interpolate the volume in the anterior-posterior direction to fill in the “missing” slices. The results are shown in Figure 9. For the reconstruction-first method, we show the reconstructed volume registered to the atlas. The reconstructed volume is very smooth. In the sagittal view, the central region seems to register with the atlas well. But it is clear that the front and back of the brain are misaligned. Because the goal of our method is to map the experimental slices to its corresponding coordinates in the atlas volume, we emphasize more on the correctness of alignment rather than smoothness. The sagittal view shows that with our method, experimental brains are positioned correctly on top of the atlas volume.

Because sectional brains only consist of about 1/7 to 1/6 of a full brain length, showing the sagittal view of these thin stacks does not exhibit the correctness of alignment. Instead, we show four evenly-spaced slices in each experimental brain and their corresponding planes in the atlas volume after we mapped them to the same coordinates. Figure 10 displays a triplet of images for each slice location. Each row shows two triplets of images. In each triplet, the left image is the registered slice using our method, the center is the corresponding atlas plane, and the right image is the registered slice using the reconstruction-first method. To give the registration-first method a fairer comparison, we try to avoid slices where the corresponding planes don't contain a full slice: we constrain our slice selection to the portion that has a close-to-full slice correspondence in the reconstructed volume. If the plane correspondence is correct, the images will show the same anatomical features. Clearly our method catches the correspondence better than the reconstruction-first method. We can also glance at the registration performance from Figure 10.

**Figure 10A:**
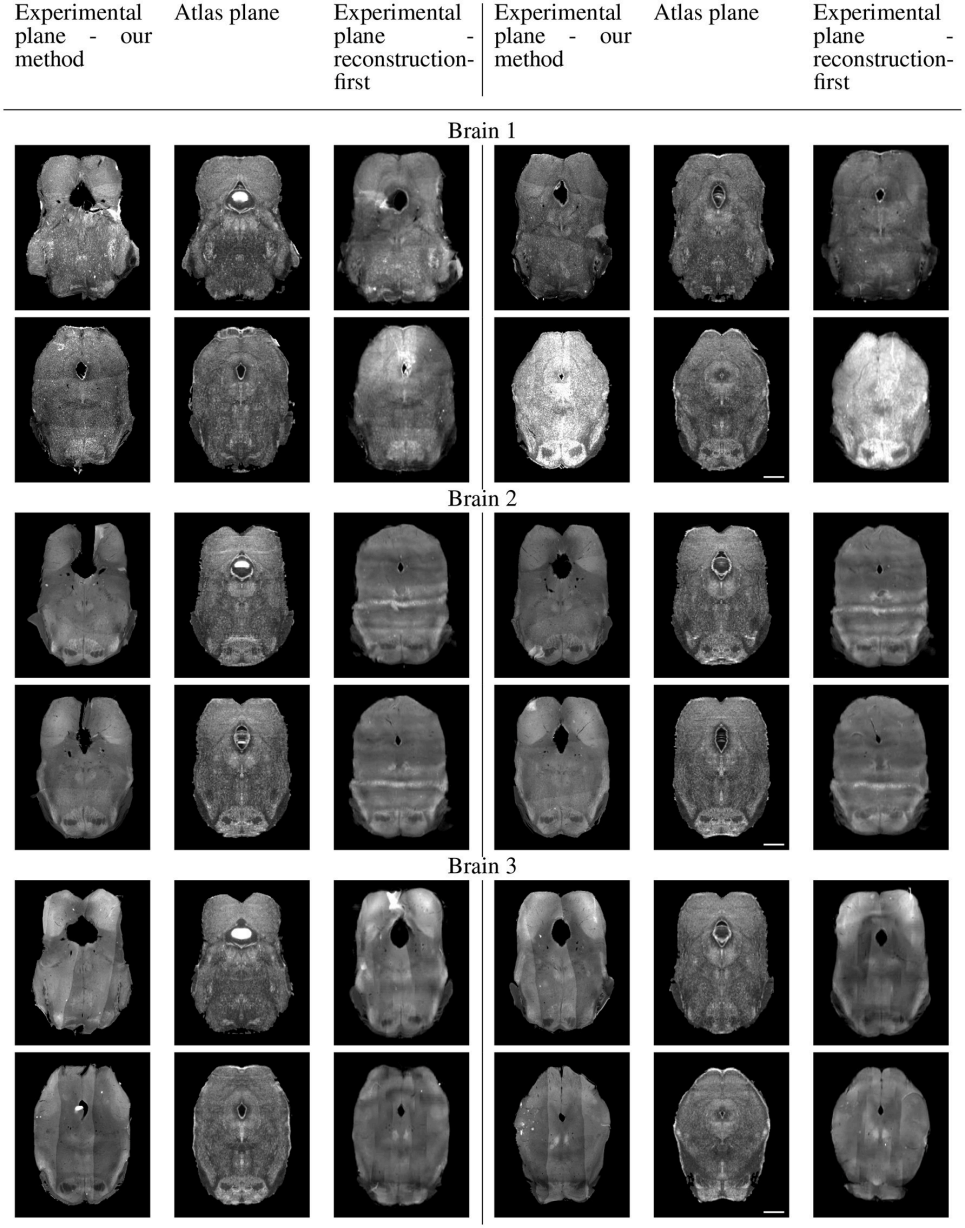
Real experiments results: experimental images and their corresponding atlas planes after registration. Intensity 2 × in all images for visualization purposes. Scale, 1 mm.

**Figure 10B:**
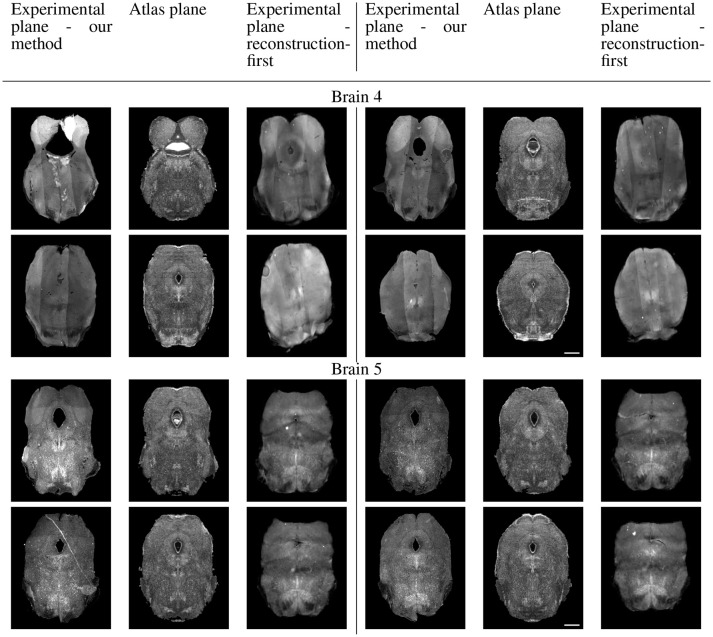
Real experiments results: experimental images and their corresponding atlas planes after registration. Intensity 3 × in experimental images, 2 × in atlas images for visualization purposes. Scale, 1 mm.

## 4. Discussion

Histological sectioning is the most commonly used method to investigate organizations of normal and diseased brains. Individual brain variations and distortions and intensity inconsistencies caused by sample preparations make aligning histological brain slices to a reference a challenging task for both experts and computer algorithms. To address these challenges, we put together a direct approach to solving the mapping problem between a 2D histological sequence and a reference volume that allows us to determine the best corresponding slice for each experimental slice before attempting any nonrigid alignment. It uses the L2 norm of HOG difference as the image comparison metric and the average matching index difference between half-images to create a sectioning angle measurement. The HOG metric enables image similarity comparison without the need of deformable registration. This produces a robust framework that leverages brain structural characteristics and symmetry to determine the cutting angle and matching slices without initial reconstruction. Avoiding reconstruction improves accuracy by preventing z-shift problems as validated by our comparison experiments. In 2D nonrigid registration, we augmented the standard MRF on medical image registration to model accumulated tension when deforming tissues to more naturally deal with the easily-deformed cavities throughout the brain. This requires us to use squared distance pairwise term and pass simulated stress across iterations.

Interestingly, the results from the comparison experiment between the reconstruction-first method and our method show that using sectional reconstruction for registration still introduces small errors. These methods must compromise between thinner sections, with less z-shift issues, and thicker sections that contain better matching information. As a result, our method has better accuracy for registrations of sections with only 1/7 of the full brain.

Since our method is mostly automatic, and the accuracy is similar to or better than an expert neuroscientist even for datasets where many slices are corrupted, we have successfully used our method to map multiple brain datasets in a recent anatomical study (Ren et al., 2018) to the ABA, making multi-brain data analysis possible and accurate. Further work should be able to improve the quality of our registration by tailoring the non-rigid deformation to emphasize salient features, and incorporating 3-D information in this step.

The ABA (2015) also contains a population average of serial two-photon (STP) tomography images. While we used the grayscale Nissl volume of the ABA in our project, because our method is very robust to intensity variation, we tested aligning a Nissl-stained experimental image to the corresponding STP plane of the ABA. The STP volume is easier to prepare because the quality of imaging is overall better. The results are promising. In fact, we get similar qualitative results as the Nissl-stained atlas slice. Clearly, while further work will be needed in this multi-modality task, it seems this method might be useful to these applications as well.

## Data Availability Statement

Code and sample dataset in this study are available at the project's website, https://sites.google.com/view/brain-mapping.

## Author Contributions

JX wrote the software to register the histological slice sequence to the ABA. JR prepared the datasets used in this paper and has been significantly involved in evaluation. MH provided important advice and oversees the entire project with LL.

### Conflict of Interest Statement

The authors declare that the research was conducted in the absence of any commercial or financial relationships that could be construed as a potential conflict of interest.
